# Isquemia e reperfusão por circulação retrógrada: estudo comparativo experimental

**DOI:** 10.1590/1677-5449.009016

**Published:** 2017

**Authors:** Cesar Roberto Busato, Carlos Alberto Lima Utrabo, Leandro Cavalcante Lipinski, Keizi Dayane de Lima, Márcio Dias Guilherme, Nicolas Brandalize Medeiros, Samela Basi Fagundes, Willman Josviak

**Affiliations:** 1 Universidade Estadual de Ponta Grossa – UEPG, Departamento de Medicina, Ponta Grossa, PR, Brasil.

**Keywords:** arterialização venosa, isquemia, reperfusão

## Abstract

**Contexto:**

Isquemia crítica de membro inferior sem leito distal tem opções restritas para tratamento. Desviar o fluxo de maneira retrógrada através da circulação venosa é alternativa amparada em evidências de inúmeros trabalhos publicados.

**Objetivos:**

Comparar o comportamento de variáveis clínicas e laboratoriais em extremidades de suínos submetidas a isquemia e a isquemia com reperfusão por circulação retrógrada entre si e em relação e a um grupo controle.

**Métodos:**

Dez suínos foram separados em dois grupos. No grupo 1 (n=5), controle, avaliaram-se padrões fisiológicos de variáveis como fluxo ao Doppler, temperatura, gasometria, lactato, creatinoquinase (CK) e pressão arterial. No grupo 2 (n=5), após um período médio de isquemia de 27 minutos e 30 segundos, consequente à interrupção do fluxo nas artérias femorais, os animais foram submetidos a arterialização venosa no membro posterior esquerdo e a manutenção da isquemia no direito. As variáveis foram analisadas separadamente durante momentos 0, 2, 3, 4 e 6 horas após a reperfusão para efeito de comparação entre si e com o grupo controle.

**Resultados:**

A análise das variáveis mostrou, em ambos os procedimentos, queda de BE e pO_2_, com elevação significativa de lactato e CK em relação ao grupo controle. Nos membros isquêmicos arterializados, encontramos fluxo ao Doppler e maiores pressões arteriais e temperaturas quando comparadas ao membro em isquemia.

**Conclusões:**

A análise comparativa das extremidades em isquemia e isquemia arterializada mostrou, em relação ao grupo controle, um quadro de acidose metabólica, com significativo aumento de lactato e CK, que sugerem dano celular e sinais de reperfusão retrógrada nas extremidades arterializadas.

## INTRODUÇÃO

Em isquemia crítica sem leito arterial distal, não é possível derivar o sangue para uma extremidade arterial pérvia distal à obstrução. Desviar o fluxo de maneira retrógrada através da circulação venosa é uma alternativa viável amparada em evidências de inúmeros trabalhos publicados^1^^-^ 13. Esse conceito baseia-se na teoria de que, na ausência de pressão arterial primária nas arteríolas, o sangue fornecido utilizando o sistema venoso distal por meio da arterialização é capaz de suprir os tecidos periféricos e fornecer oxigenação adequada^3^^-^ 5 ^,^ 13.

Diversas variáveis têm sido utilizadas para a avaliação de hipóxia tecidual em modelos animais, como avaliação do fluxo arterial através de aparelho Doppler^14^^,^ 15, aferição da temperatura^16^, gasometria^17^, dosagem de lactato^18^, creatinoquinase (CK)^19^ e medidas de pressão arterial de extremidade^20^. O objetivo deste trabalho é comparar, o comportamento dessas variáveis clínicas e laboratoriais em extremidades de suínos, submetidas a isquemia e, a reperfusão por circulação retrógrada entre si e em relação a um grupo controle.

## MÉTODOS

Este projeto foi aprovado pelo Comitê de Ética em Pesquisa Animal (CEUA 009/2013) e realizado no Laboratório de Técnica Operatória e Cirurgia Experimental, Faculdade de Medicina, Universidade Estadual de Ponta Grossa (UEPG). Foram utilizados 10 suínos cruzados Large White-Landrace, divididos em dois grupos. No grupo 1 (n=5), controle, foi realizada avaliação dos padrões fisiológicos de qualidade de fluxo arterial pelo aparelho Doppler, temperatura, gasometria arterial (pH, excesso de base, bicarbonato, pressão parcial de oxigênio e pressão parcial de dióxido de carbono), lactato, CK e pressão arterial em extremidades posteriores, através de dissecção arterial femoral. As determinações foram realizadas em animais destinados à aula prática de Técnica Operatória antes dos procedimentos cirúrgicos.

No grupo 2 (n=5), com intervenção, as variáveis pesquisadas no grupo 1 foram determinadas em membro posterior esquerdo isquêmico arterializado e em membro isquêmico no direito.

Os animais de ambos os grupos receberam medicação pré-anestésica com ketamina (14 mg/kg), xilazina (0,2 mg/kg) e acepromazina (0,4 mg/kg). Foram induzidos à anestesia com propofol (5 mg/kg) e mantidos em anestesia inalatória com isofluorano em concentração alveolar mínima de 1,2 a 1,7%.

Em todos os membros posteriores estudados, foram realizadas dissecções de artérias e veias femorais comuns. Os vasos dos membros controle (grupo 1) foram utilizados somente como fonte de coleta. Nos membros dos animais do grupo 2, as veias foram utilizadas para coleta de sangue por punção direta e, através de arteriotomias, as extremidades distais e proximais das artérias femorais comuns foram canalizadas com cateter intravascular nº 14, (cateter intravenoso ESCALPE sem ponta de entrada, com parede de Teflon Esterilizado por Óxido de Etileno; SOLIDOR®), ligadas e ocluídas, iniciando-se a medida do tempo de isquemia.

Nos membros isquêmicos arterializados, a veia safena externa (parva) foi dissecada. Após anticoagulação sistêmica com 5.000 UI de heparina, realizou-se ligadura proximal, venotomia e rotura das válvulas a jusante com auxílio de valvulótomo de Lengua. Dilatação distal com soro heparinizado através de sonda nº 4. Canalização e fixação com cateter intravascular nº 14 (SOLIDOR®). Procedeu-se à conexão das extremidades proximais das artérias femorais comum com as veias safenas utilizando-se um cateter de silicone (EXTENSOR para Cateter Reversível Luer Lock 20 cm, 2 conectores macho – 10F; HARTMANN®), iniciando-se a contagem do tempo de arterialização T0.

**Figura 1:**
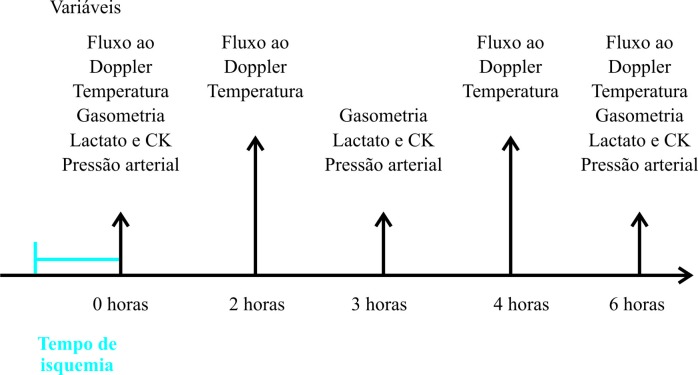
Determinação das variáveis estudadas nos membros isquêmicos e isquêmicos arterializados ao longo do tempo da pesquisa.

Após o término dos procedimentos cirúrgicos e durante os tempos 0, 2, 4 e 6 horas de reperfusão, respectivamente momentos T0, T1, T3 e T4 (Figura 1), ao longo da investigação, realizou-se pesquisa da presença de fluxo sanguíneo nas extremidades com auxílio de um aparelho Doppler portátil (Doppler Vascular DV 600; Martec®) e aferição da temperatura com termômetro infravermelho (termômetro infravermelho Sem Contato FR1DZ1; G-TECH®). O fluxo sanguíneo foi avaliado na artéria safena, em cada membro. As aferições de temperatura foram realizadas, em três tempos, em um ponto fixo no espaço interdigital na região plantar e, na região dorsal, em três pontos distantes um do outro cerca de 3 cm ao longo da extremidade de cada membro. A distância entre o termômetro e a pele foi de aproximadamente 3 a 5 cm.

Nos tempos 0, 3 e 6 horas de reperfusão, respectivamente momentos T0, T2 e T4 (Figura 1), colheram-se, com seringas heparinizadas de 3 mL, amostras de sangue para gasometria, através dos cateteres das extremidades distais das arteriotomias (femoral superficial); por punção direta das femorais, colheu-se sangue venoso para dosagem de lactato e CK; e, utilizando-se régua e equipo em coluna de soro fisiológico, determinaram-se as pressões nas extremidades arteriais distais (femoral superficial) e proximais (femoral comum).

Procedeu-se à administração de 5.000 UI de heparina a cada 3 horas durante o transcorrer da pesquisa.

Ao final dos procedimentos, os animais do grupo 2 foram submetidos a eutanásia conforme resolução 1000/2012 do Conselho Federal de Medicina Veterinária (CFMV).

### Análise estatística

Os resultados foram submetidos a análise quanto à distribuição de normalidade de acordo com o teste de Anderson Darling. O teste qui-quadrado com correção de Yates foi utilizado para variáveis qualitativas, e o teste *t* de Student para variáveis quantitativas, sendo estatisticamente significativo p < 0,05.

## RESULTADOS

As variáveis pesquisadas em membros isquêmicos arterializados, com tempo médio de isquemia de 27 minutos e 30 segundos, foram comparadas às determinadas em membros isquêmicos e do grupo controle.

### Fluxo arterial

A pesquisa de fluxo arterial ao Doppler nos membros isquêmicos arterializados mostrou, em T0, ausência de fluxo em um membro, padrão venoso pulsátil em três e pulsátil arterial em um; em T1, os quatro membros com fluxo apresentavam padrão venoso pulsátil; em T3, observou-se padrão pulsátil arterial em um e venoso pulsátil em três; em T4, três membros apresentavam padrão pulsátil venoso e dois, ausência de fluxo. Os membros isquêmicos apresentaram ausência de fluxo ao longo da pesquisa.

Foram considerados para estudo apenas os dados referentes aos membros isquêmicos arterializados dos animais que apresentavam fluxo ao Doppler no momento da coleta ou da determinação: quatro animais até o momento T3 e três animais no momento T4.

### Temperatura

Embora não tenha havido controle ambiental, as médias de variação da temperatura, nas extremidades estudadas, apresentaram curvas semelhantes, com médias significativamente inferiores às obtidas no grupo controle ao longo do tempo (Tabela 1). Quando comparadas entre si, as médias das temperaturas dos membros isquêmicos arterializados apresentavam uma diferença não significativa (p= 0,55) de 2,25 °C a mais que as dos os membros isquêmicos no momento T4 (Tabela 2).

**Tabela 1:** Comparação da temperatura de membros arterializados e em isquemia em relação ao grupo controle nos diferentes momentos.

**Temperatura (momentos)**	**Arterialização**	**Controle**	**p**	**Isquemia**	**Controle**	**p**
	**Média (desvio padrão)**	**Média (desvio padrão)**		**Média (desvio padrão)**	**Média (desvio padrão)**	
T0	29,81 (3,77)	35,65 (0,55)	0,01	30,37 (3,97)	35,65 (0,55)	0,02
T1	26,34 (4,46)	35,65 (0,55)	0,002	26,32 (3,45)	35,65 (0,55)	0,0005
T3	24,77 (2,62)	35,65 (0,55)	0,0001	24,70 (2,35)	35,65 (0,55)	0,0001
T4	26,74 (5,67)	35,65 (0,55)	0,010	24,49 (2,20)	35,65 (0,55)	0,0001

Teste *t* de Student.

**Tabela 2:** Comparação da temperatura de membros isquêmicos arterializados e em isquemia nos diferentes momentos.

**Temperatura (momentos)**	**Arterialização**	**Isquemia**	**p**
	**Média (desvio padrão)**	**Média (desvio padrão)**	
T0	29,81 (3,77)	30,37 (3,97)	0,84
T1	26,34 (4,46)	26,32 (3,45)	0,99
T3	24,77 (2,62)	24,70 (2,35)	0,97
T4	26,74 (5,67)	24,49 (2,20)	0,55

Teste *t* de Student.

### Gasometria

O pH dos membros isquêmicos arterializados e em isquemia partiu de níveis levemente superiores aos do grupo controle e apresentou curvas semelhantes, com queda não significativa em T2, um pouco mais acentuada do que em T4 (Tabelas 3, 4 e 5). Quando comparados entre si, não apresentaram significância (Tabelas 6, 7 e 8).

**Tabela 3:** Comparação das variáveis bioquímicas e de pressão arterial em membros arterializados e em isquemia em relação ao grupo controle no momento T0.

**Variáveis**	**Arterialização**	**Controle**	**p**	**Isquemia**	**Controle**	**p**
	**Média (desvio padrão)**	**Média (desvio padrão)**		**Média (desvio padrão)**	**Média (desvio padrão)**	
pH	7,32 (0,11)	7,30 (0,15)	0,83	7,31 (0,12)	7,30 (0,15)	0,92
BE	2,68 (1,02)	2,38 (2,47)	0,83	2,18 (1,38)	2,38 (2,47)	0,89
HCO_3_^-^	29,45 (1,77)	30,62 (4,17)	0,62	29,15 (2,29)	30,62 (4,17)	0,55
pO_2_	166,33 (61,91)	298,10 (128,32)	0,10	158,93 (58,34)	298,10 (128,32)	0,09
pCO_2_	60,75 (16,19)	68,82 (34,49)	0,68	60,60 (17,63)	68,82 (34,49)	0,68
Lactato	18,55 (2,99)	17,14 (6,96)	0,72	21,20 (10,28)	17,14 (6,96)	0,50
CK	1.647,50 (590,89)	940,00 (364,18)	0,06	1.612,50 (563,68)	940,00 (364,18)	0,07
Pressão arterial	55,75 (15,76)	35,86 (22,86)	0,18	47,50 (22,49)	35,86 (22,86)	0,47

**Tabela 4:** Comparação das variáveis bioquímicas e de pressão arterial em membros arterializados e em isquemia em relação ao grupo controle no momento T2.

**Variáveis**	**Arterialização**	**Controle**	**p**	**Isquemia**	**Controle**	**p**
	**Média (desvio padrão)**	**Média (desvio padrão)**		**Média (desvio padrão)**	**Média (desvio padrão)**	
pH	7,26 (0,08)	7,30 (0,15)	0,65	7,25 (0,06)	7,30 (0,15)	0,55
BE	1,13 (1,97)	2,38 (2,47)	0,44	0,68 (1,41)	2,38 (2,47)	0,26
HCO_3_^-^	29,30 (1,47)	30,62 (4,17)	0,57	29,08 (1,28)	30,62 (4,17)	0,50
pO_2_	143,65 (89,98)	298,10 (128,32)	0,08	167,53 (143,26)	298,10 (128,32)	0,19
pCO_2_	57,80 (23,85)	68,82 (34,49)	0,61	68,40 (11,02)	68,82 (34,49)	0,98
Lactato	40,30 (19,38)	17,14 (6,96)	0,04	36,10 (21,17)	17,14 (6,96)	0,10
CK	1.747,00 (556,34)	940,00 (364,18)	0,03	1.704,25 (554,79)	940,00 (364,18)	0,04
Pressão arterial	42,63 (19,61)	35,86 (22,86)	0,65	37,75 (15,76)	35,86 (22,86)	0,89

Teste *t* de Student.

**Tabela 5:** Comparação das variáveis bioquímicas e de pressão arterial em membros arterializados e em isquemia em relação ao grupo controle no momento T4.

**Variáveis**	**Arterialização**	**Controle**	**p**	**Isquemia**	**Controle**	**p**
	**Média (desvio padrão)**	**Média (desvio padrão)**		**Média (desvio padrão)**	**Média (desvio padrão)**	
pH	7,21 (0,12)	7,30 (0,15)	0,41	7,20 (0,09)	7,30 (0,15)	0,34
BE	-0,50 (3,58)	2,38 (2,47)	0,41	1,07 (2,72)	2,38 (2,47)	0,11
HCO_3_^-^	29,07 (1,61)	30,62 (4,17)	0,57	27,87 (2,48)	30,62 (4,17)	0,35
pO_2_	95,83 (13,82)	298,10 (128,32)	0,04	94,67 (3,89)	298,10 (128,32)	0,04
pCO_2_	76,23 (21,75)	68,82 (34,49)	0,75	71,87 (15,09)	68,82 (34,49)	0,89
Lactato	61,93 (19,70)	17,14 (6,96)	0,003	55,33 (19,15)	17,14 (6,96)	0,006
CK	2.020,67 (621,81)	940,00 (364,18)	0,02	1.879,33 (425,96)	940,00 (364,18)	0,02
Pressão arterial	33,17 (19,20)	35,86 (22,86)	0,87	36,67 (13,58)	35,86 (22,86)	0,96

Teste *t* de Student.

**Tabela 6:** Comparação das variáveis bioquímicas e de pressão arterial de membros isquêmicos arterializados e em isquemia no momento T0.

**Variáveis**	**Arterialização**	**Isquemia**	**p**
	**Média (desvio padrão)**	**Média (desvio padrão)**	
pH	7,32 (0,11)	7,31 (0,12)	0,91
BE	2,68 (1,02)	2,18 (1,38)	0,58
HCO_3_^-^	29,45 (1,77)	29,15 (2,29)	0,84
pO_2_	166,33 (61,91)	158,93 (58,34)	0,87
pCO_2_	60,75 (16,19)	60,60 (17,63)	0,99
Lactato	18,55 (2,99)	21,20 (10,28)	0,64
CK	1.647,50 (590,89)	1.612,50 (563,68)	0,93
Pressão arterial	55,75 (15,76)	47,50 (22,49)	0,57

Teste *t* de Student.

**Tabela 7:** Comparação das variáveis bioquímicas e de pressão arterial de membros isquêmicos arterializados e em isquemia no momento T2.

**Variáveis**	**Arterialização**	**Isquemia**	**p**
	**Média (desvio padrão)**	**Média (desvio padrão)**	
pH	7,26 (0,08)	7,25 (0,06)	0,85
BE	1,13 (1,97)	0,68 (1,41)	0,72
HCO_3_^-^	29,30 (1,47)	29,08 (1,28)	0,83
pO_2_	143,65 (89,98)	167,53 (143,26)	0,79
pCO_2_	57,80 (23,85)	68,40 (11,02)	0,45
Lactato	40,30 (19,38)	36,10 (21,17)	0,78
CK	1.747,00 (556,34)	1.704,25 (554,79)	0,92
Pressão arterial	42,63 (19,61)	37,75 (15,76)	0,71

Teste t de Student.

**Tabela 8:** Comparação das variáveis bioquímicas e de pressão arterial de membros isquêmicos arterializados e em isquemia no momento T4.

**Variáveis**	**Arterialização**	**Isquemia**	**p**
	**Média (desvio padrão)**	**Média (desvio padrão)**	
pH	7,21 (0,12)	7,20 (0,09)	0,91
BE	0,50 (3,58)	1,07 (2,72)	0,58
HCO_3_^-^	29,07 (1,61)	27,87 (2,48)	0,52
pO_2_	95,83 (13,82)	94,67 (3,89)	0,89
pCO_2_	76,23 (21,75)	71,87 (15,09)	0,79
Lactato	61,93 (19,70)	55,33 (19,15)	0,70
CK	2.020,67 (621,81)	1.879,33 (425,96)	0,76
Pressão arterial	33,17 (19,20)	36,67 (13,58)	0,81

Teste *t* de Student.

### Excesso de base

Tanto os membros isquêmicos arterializados quanto aqueles em isquemia apresentaram uma queda progressiva do excesso de base (BE) em relação ao grupo controle, mais acentuada naqueles em isquemia (Tabelas 3, 4 e 5). Quando comparados entre si apresentavam uma diferença não significativa (p=0,58) de 1,57 em T4 (Tabelas 6, 7 e 8).

### Concentração de bicarbonato

Tanto os membros isquêmicos arterializados quanto aqueles em isquemia apresentaram uma queda progressiva da concentração de bicarbonato (HCO_3_^-^), mais acentuada em isquemia, especialmente no momento T4 (Tabelas 3, 4 e 5), mas sem significância tanto em relação ao grupo controle quanto entre si (Tabelas 6, 7 e 8).

### Pressão parcial de oxigênio

As pressões parciais de oxigênio (pO_2_) nos membros isquêmicos arterializados e em isquemia partiram de níveis menores que os do grupo controle e apresentaram curvas semelhantes, com quedas significativas em T4 (Tabelas 3, 4 e 5). Comparadas entre si, não apresentaram significância (Tabelas 6, 7 e 8).

### Pressão parcial de dióxido de carbono

As pressões parciais de dióxido de carbono (pCO_2_) mostraram valores semelhantes e inferiores ao grupo controle no momento T0 com aumento progressivo da média dos membros em isquemia e leve queda na média daqueles em isquemia com arterialização em T2. Embora as médias de ambos os grupos tenham mostrado aumento em T4, esse aumento foi maior no grupo isquemia com arterialização (Tabelas 3, 4 e 5). Comparadas entre si, as médias não apresentaram significância (Tabelas 6, 7 e 8).

### Lactato

As médias dos valores de lactato partiram de nível próximo ao do grupo controle e apresentaram curvas semelhantes, com aumento progressivo em T2, significativo para membros isquêmicos arterializados, e mais acentuado e significativo tanto para arterialização quanto para isquemia no momento T4 (Tabelas 3, 4 e 5). Comparadas entre si, as médias não apresentaram significância (Tabelas 6, 7 e 8).

### Creatinoquinase

As médias dos valores de CK partiram de níveis superiores aos do grupo controle, mostrando aumento significativo tanto para membros isquêmicos arterializados quanto para isquemia em T2 e T4 (Tabelas 3, 4 e 5). No entanto, as médias não apresentaram significância entre si (Tabelas 6, 7 e 8).

### Pressão arterial

Para efeito de cálculo, foram consideradas as razões de pressões arteriais distais pelas proximais. Nos membros isquêmicos arterializados, a média dessas razões partiu de nível superior ao do grupo controle e ao dos membros em isquemia. As médias de ambos os grupos com intervenção apresentaram queda em T2, embora os membros isquêmicos arterializados tenham permanecido em nível mais elevado. Em T4, as médias se aproximaram às do grupo controle (Tabelas 3, 4 e 5) e não apresentaram diferença significativa entre si (Tabelas 6, 7 e 8).

As curvas de valor absoluto das pressões distais apresentaram padrão semelhante às razões, igualmente sem significância.

## DISCUSSÃO

Não existem trabalhos experimentais de isquemia e reperfusão (I/R) por arterialização venosa que tenham testado as variáveis estudadas em animais. Nosso modelo produz uma isquemia aguda diferente da crônica, em que o tempo em isquemia sem necrose pode levar à produção de estímulos para arteriogênese.

Embora os procedimentos tenham sido realizados no mesmo animal, em ambos os membros posteriores, as variáveis estudadas foram determinadas separadamente. A utilização do membro contralateral é frequente em estudos de isquemia unilateral^21^^,^ 22, pois permite que as medidas sejam realizadas sobre o mesmo substrato, embora não se possa afastar totalmente a interferência de um membro sobre o contralateral. O modelo experimental utilizado no presente estudo reproduz situações de isquemia e reperfusão por arterialização venosa com passagem retrógrada do fluxo venoso arterializado com maior pressão (arterial proximal) para o leito arterial de menor pressão (artéria distal)^13^.

Foram desprezados os valores determinados no membro isquêmico arterializado quando não havia fluxo ao Doppler na extremidade.

A presença de fluxo ao Doppler mostrou padrão pulsátil arterial e venoso pulsátil, este também encontrado em fístulas arteriovenosas^23^.

Sasajima et al., em estudo de arterialização venosa profunda em camundongos, mostraram que a rotura das válvulas ao nível de veia femoral era acompanhada de aumento de temperatura cutânea em região coxofemoral e articulação do joelho. Mostraram ainda que a hipertermia em extremidade distal só ocorria quando as válvulas de veia poplítea eram rompidas^10^.

O aumento da temperatura média dos membros isquêmicos arterializados, observado a partir do momento T3, sugere perviedade do sistema e corrobora a utilidade da valvulotomia na veia arterializada.

Durante a reperfusão, o edema tecidual acaba se instalando com potencial agravante da injúria tecidual e da resposta sistêmica. No contexto da I/R, alterações locais e sistêmicas se desenvolvem em diversos sistemas: endotelial, sanguíneo, metabólico, ácido-base, etc.^20^.

Szokoly et al. demonstraram, em ratos submetidos a I/R de membros posteriores, uma queda continua e significativa do pH venoso comparado ao basal na primeira hora. Essa mudança foi acompanhada de alterações na pCO_2_ e na pO_2_, que apresentaram sinais moderados de compensação respiratória^20^.

Mondek et al. mostraram um estudo piloto em que o grau máximo de acidose ocorreu 2 horas após o início da reperfusão em membro isquêmico devido a clampeamento vascular. A amostra de sangue foi colhida na veia femoral ipsilateral^24^.

Achados deste estudo mostram um quadro de acidose metabólica, com queda no valor de BE e pouca alteração em HCO_3_^-^. A queda de BE sugere tamponamento, o que permite certa estabilidade nos valores do pH. As variações na pCO_2_ e pO_2_ foram inespecíficas, apresentando queda significativa da pO_2_ em ambos os membros acompanhada de aumento da pCO_2_ ao final do experimento. Esses achados são compatíveis com outros estudos sobre I/R^20^^,^ 25.

Os trabalhos de Szokoly et al. e Mondek et al. avaliaram o pH na veia coletora do membro, visto que a reperfusão se deu por via arterial^20^^,^ 24. Em nossa pesquisa, determinamos a gasometria na extremidade distal da artéria femoral, uma vez que a reperfusão se deu por via venosa, e nossa intenção foi avaliar o fluxo retrógrado.

Sako et al., durante cirurgia de aneurisma de aorta abdominal que envolve I/R de extremidades, evidenciaram aumento transitório de lactato e redução de pH em veias ilíacas pós-reperfusão^26^.

Theodoraki et al., em estudo sobre gradiente trans-hepático de lactato durante I/R em hepatectomias, encontraram aumento na produção de lactato hepático, observado 50 minutos após a reperfusão. Mostraram ainda correlação positiva entre os níveis de lactato intraoperatório sistêmico e o gradiente trans-hepático de lactato, sugerindo uma significativa contribuição da reperfusão hepática ao estado de hiperlactatemia sistêmica^27^.

As médias de valores de lactato encontradas neste estudo partiram de nível semelhante ao do grupo controle e apresentaram curvas semelhantes, com aumento progressivo significativo e mais acentuado nos membros isquêmicos arterializados, nos quais ocorreu reperfusão por via retrógrada (fluxo presente ao Doppler).

Woodruff et al., em estudo para avaliar a capacidade de um fármaco contra a injúria provocada pela isquemia e subsequente reperfusão (I/R), demonstraram a elevação de CK no grupo submetido a I/R, o que não foi observado no grupo submetido somente a isquemia^28^. Isso pode sugerir que a base patogênica para a elevação do marcador de injúria muscular seria a reperfusão após isquemia.

Nesta pesquisa, os valores de CK apresentaram comportamento semelhante ao do lactato, mostrando aumentos significativos em T2 e ainda mais acentuados em T4, especialmente nos membros isquêmicos arterializados.

Szokoly et al., ao estudar I/R em membros posteriores de camundongos, encontrou queda da pressão arterial média em torno de 20% após a reperfusão. Possíveis eventos compensatórios paralelos ao procedimento ou, até mesmo, à vasodilatação devido à reperfusão do membro podem explicar a queda dos valores de pressão^20^.

A média das razões de pressões arteriais dos membros isquêmicos arterializados partiu de nível superior ao do grupo controle e dos membros em isquemia. As médias de ambos os grupos com intervenção apresentaram queda em T2, embora os isquêmicos arterializados tenham permanecido em nível mais elevado. Em T4, as médias se aproximaram às do grupo controle. A curva de queda da pressão arterial distal foi semelhante à curva das razões entre a pressão arterial distal e proximal, o que não sugere a interferência da pressão arterial proximal nos valores encontrados.

## CONCLUSÃO

Os achados desta pesquisa são compatíveis com quadros de acidose metabólica, com significativo aumento de CK e lactato, sugerindo dano celular em ambas as extremidades e sinais de reperfusão retrógrada, devido à manutenção da presença do fluxo ao Doppler nos membros isquêmicos arterializados.

## References

[1] Alexandrescu V, Ngongang C, Vincent G, Ledent G, Hubermont G (2011). Deep calf veins arterialization for inferior limb preservation in diabetic patients with extended ischaemic wounds, unfit for direct arterial reconstruction: preliminary results according to an angiosome model of perfusion. Cardiovasc Revasc Med.

[2] Djoric P (2011). Early individual experience with distal venous arterialization as a lower limb salvage procedure. Am Surg.

[3] Busato CR, Utrabo CA, Gomes RZ (2010). Utilização da safena magna in situ para arterialização do arco venoso do pé. J Vasc Bras.

[4] Taylor RS, Belli AM, Jacob S (1999). Distal venous arterialization for salvage of critically ischaemic inoperable limbs. Lancet.

[5] Mutirangura P, Ruangsetakit C, Wongwanit C, Sermsathanasawadi N, Chinsakchai K (2011). Pedal bypass with deep venous arterialization: the therapeutic option in critical limb ischemia and unreconstructable distal arteries. Vascular.

[6] Lengua F, Madrid A, Acosta C, Vargas J (2010). Arterializacion venosa temporal del pie diabético. J Vasc Bras.

[7] Lu XW, Idu MM, Ubbink DT, Legemate DA (2006). Meta-analysis of the clinical effectiveness of venous arterialization for salvage of critically ischaemic limbs. Eur J Vasc Endovasc Surg.

[8] Özbek C, Kestelli M, Emrecan B (2005). A novel approach: ascending venous arterialization for atherosclerosis obliterans. Eur J Vasc Endovasc Surg.

[9] Schreve MA, Minnee RC, Bosma J, Leijdekkers VJ, Idu MM, Vahl AC (2014). Comparative study of venous arterialization and pedal bypass in a patient cohort with critical limb ischemia. Ann Vasc Surg.

[10] Sasajima T, Kikuchi S, Ishikawa N, Koyama T (2014). Skin temperature in lower hind limb subjected to distal vein arterialization in rats. Adv Exp Med Biol.

[11] Houlind K, Christensen J, Hallenberg C, Jepsen JM (2013). Early results from an angiosome-directed open surgical technique for venous arterialization in patients with critical lower limb ischemia. Diabet Foot Ankle.

[12] Ozbek C, Kestelli M, Bozok S (2014). Surgical stimulation of angiogenesis. Asian Cardiovasc Thorac Ann.

[13] Busato CR, Utrabo CA, Lipinski LC (2016). Experimental model for the study of retrograde flow. J Vasc Bras.

[14] Bordinhão A (2013). Comparação entre a Dopplermetria e o fluxo livre da artéria torácica interna de cães com e sem o uso de noradrenalina. Rev Bras Cir Cardiovasc.

[15] Poerschke RA, Silveira DA, Lodi P, Titton W, Marx G, Lampert AS (2012). Temporary vascularization on ischemic limbs through arterial-medular shunt: an experimental work. J Vasc Bras.

[16] Brioschi ML, Mehl A, Oliveira AG (2007). Exame de termometria cutânea infravermelha na avaliação do pé diabético. Rev Méd Paraná.

[17] Hurtado Rojas P, Alves Tannous L, Von Bahten LC, Castro Villegas F, Gasparetto J (2013). Análise da gasometria e dos niveis de lactato na hipertensão intra-abdominal associada à sepse abdominal: Modelo experimental em ratos. Panamerican J Trauma.

[18] Nagy O, Seidel H, Paulíková I, Mudron P, Kovác G (2006). Use of blood gases and lactic acid analyses in diagnosis and prognosis of respiratory diseases in calves. Bull Vet Inst Pulawy.

[19] Currie IS, Wakelin SJ, Lee AJ, Chalmers RT (2007). Plasma creatine kinase indicates major amputation or limb preservation in acute lower limb ischemia. J Vasc Surg.

[20] Szokoly M, Nemeth N, Hamar J, Furka I, Miko I (2006). Early systemic effects of hind limb ischemia-reperfusion on hemodynamics and acid-base balance in the rat. Microsurgery.

[21] Thaveau F, Zoll J, Bouitbir J (2009). Contralateral leg as a control during skeletal muscle ischemia-reperfusion. J Surg Res.

[22] Mansour Z, Bouitbir J, Charles AL (2012). Remote and local ischemic preconditioning equivalently protects rat skeletal muscle mitochondrial function during experimental aortic cross-clamping. J Vasc Surg.

[23] Barros FS, Pontes SM, Silva WP, Prezotti BB, Sandri JL (2006). Identificação pelo Doppler colorido de fístula arteriovenosa na trombose venosa profunda. J Vasc Bras.

[24] Mondek P, Sefranek V, Tomka J (2002). Regional biochemical and hematologic changes in patients after revascularization of the lower extremities in ischemia of the extremities. Rozhl Chir.

[25] Tejchman K, Domanski L, Sienko J (2006). Early acid-base balance disorders during kidney transplantation. Trans Proc.

[26] Sako H, Hadama T, Miyamoto S (2004). Limb ischemia and reperfusion during abdominal aortic aneurysm surgery. Surg Today.

[27] Theodoraki K, Arkadopoulos N, Fragulidis G (2006). Transhepatic lactate gradient in relation to liver ischemia/reperfusion injury during major hepatectomies. Liver Transpl.

[28] Woodruff TM, Arumugam TV, Shiels IA, Reid RC, Fairlie DP, Taylor SM (2004). Protective effects of potent C5a receptor antagonist on experimental acute limb ischemia-reperfusion in rats. J Surg Res.

