# Supplementary material for: Isquemia e reperfusão por circulação retrógrada: estudo comparativo experimental
Source: J Vasc Bras. 2017 Jul-Sep;16(3):187–94. [Article in Portuguese] doi: 10.1590/1677-5449.009016 (PMC5868933; doi:10.1590/1677-5449.009016)
Supplement: Translation

# Translation: Ischemia and reperfusion by retrograde flow: experimental comparative study

**Authors:** Cesar Roberto Busato, Carlos Alberto Lima Utrabo, Leandro Cavalcante Lipinski, Keizi Dayane de Lima, Márcio Dias Guilherme, Nicolas Brandalize Medeiros, Samela Basi Fagundes, Willman Josviak

**Affiliations:** 1 Universidade Estadual de Ponta Grossa – UEPG, Departamento de Medicina, Ponta Grossa, PR, Brazil.

**Keywords:** venous arterialization, ischemia, reperfusion

## Abstract

**Background:**

There are few options for treating critical ischemia in limbs with no distal patency. Diverting flow through the venous circulation is an option supported by evidence from numerous published studies.

**Objectives:**

To compare the behavior of clinical and laboratory variables between the hind limbs of pigs subjected to ischemia and to ischemia with reperfusion by retrograde circulation and between these intervention groups and a control group.

**Methods:**

Ten pigs were divided into 2 groups. In group 1 (n=5), controls, patterns of physiological variables such as flow according to Doppler ultrasound, temperature, blood gas analysis results, lactate, creatine kinase, and blood pressure were evaluated. In group 2 (n=5), after an initial ischemia period with mean duration of 27 minutes and 30 seconds, provoked by interrupting flow through the femoral arteries, the animals were subjected to venous arterialization of the left hind limb while the right hind limb was maintained in ischemia. Variables were analyzed separately for each hind leg at 0, 2, 3, 4, and 6 hours after reperfusion and compared against each other and the control group.

**Results:**

Analysis of variables from both procedures showed decreases in BE and PO_2_ and significant increases in lactate and creatine kinase, in relation to the control group. In arterialized ischemic limbs, we observed flow on Doppler ultrasound, and arterial pressures and temperatures were higher than in the ischemic limbs.

**Conclusions:**

Comparative analysis of the extremities in ischemia and arterialized ischemia showed, in relation to the control group, metabolic acidosis with significant increases in lactate and creatine kinase, suggesting cellular damage, and there were signs of retrograde reperfusion in arterialized extremities.

## INTRODUCTION

In cases of critical ischemia in which distal arterial bed patency is lacking, it is not possible to shunt blood to an arterial extremity distal of the obstruction. Diverting flow in a retrograde direction through the venous circulation is a feasible alternative that is supported by evidence from countless published studies.^1^^-^ 13 The concept is based on the theory that in the absence of primary arterial blood pressure in arterioles, blood supplied by arterialization of the distal venous system is capable of supplying the peripheral tissues and providing adequate oxygenation.^3^^-^ 5 ^,^ 13

Many different variables have been used to evaluate tissue hypoxia in animal models, such as assessment of arterial flow using Doppler ultrasound (US),^14^^,^ 15 temperature measurements,^16^ blood gas analysis,^17^ lactate^18^ and creatine phosphokinase (CP) assays,^19^ and measurements of arterial blood pressure at extremities.^20^ The objective of this study is to compare the behavior of these clinical and laboratory variables in pigs’ hind limbs after induction of ischemia, with and without reperfusion via retrograde circulation, against each other and in relation to a control group.

## METHODS

This project was approved by the Animal Research Ethics Committee (CEUA 009/2013) and conducted in the Operating Techniques and Experimental Surgery Laboratory at the Universidade Estadual de Ponta Grossa (UEPG) Medical Faculty, Brazil. Ten Large White-Landrace cross pigs were acquired and divided into two groups. In group 1 (n=5), the control group, analyses were conducted of physiological indicators of the quality of arterial flow using Doppler US, temperature, arterial blood gas analysis (pH, base excess, bicarbonate, partial oxygen pressure and partial carbon dioxide pressure), lactate, CP, and arterial blood pressure in the hind limbs, by femoral artery dissection. These analyses were all performed on animals that had been selected for use in practical Operating Technique classes before surgical procedures were conducted.

In group 2 (n=5), the intervention group, the same variables measured in group 1 were determined for the right hind limb after induction of ischemia and the left hind limb after induction of ischemia and arterialization.

The animals in both groups were premedicated with ketamine (14 mg/kg), xylazine (0.2 mg/kg), and acepromazine (0.4 mg/kg). Anesthesia was induced with propofol (5 mg/kg) and maintained with inhaled isoflurane at a minimum alveolar concentration of 1.2 to 1.7%.

In all hind limbs studied, common femoral arteries and veins were dissected. In limbs in the control group (group 1) these vessels were only used for data collection. In the hind limbs of animals in group 2, veins were used to draw blood samples by direct puncture, while arteriotomies were performed at the distal and proximal extremities of the common femoral arteries and cannulated with number 14 intravascular catheters, (ESCALPE intravenous catheter without entry tip, with Teflon wall sterilized by ethylene oxide; SOLIDOR®), ligated, and occluded, when ischemia time measurement was initiated.

In limbs designated for arterialization after ischemia, the external (small) saphenous vein was dissected. Systemic anticoagulation was administered with 5,000 UI of heparin, followed by proximal ligature, venotomy, and rupture of downstream valves using a Lengua valvulotome, and then distal dilatation with heparinized saline via the nº 4 probe, before cannulation and fixation with the number 14 intravascular catheter (SOLIDOR®). The proximal extremities of the common femoral arteries were connected to the saphenous veins using a silicone catheter (EXTENSOR for 20 cm Luer Lock Reversible Catheter, 2 10F male connectors–; HARTMANN®), which was defined as the start of arterialization, T0.

Once the surgical procedures had been completed, at 0, 2, 4, and 6 hours of reperfusion, respectively times T0, T1, T3 and T4 (Figure 1), tests were performed to detect blood flow at the extremities with a portable Doppler US unit (DV 600 Vascular Doppler; Martec®), temperature was measured with an infrared thermometer (FR1DZ1 non-contact infrared thermometer; G-TECH®). Blood flow was tested at the saphenous artery in each limb. Temperature measurements were performed at three different times, at a fixed point in the interdigital space of the plantar region and, on the dorsal surface at three points around 3 cm apart from each other along the extremity of each limb. The distance between the thermometer and the skin was approximately 3 to 5 cm.

**Figure 1:** Determination of variables studied in ischemic limbs and arterialized ischemic limbs over duration of study.

At 0, 3, and 6 hours of reperfusion, times T0, T2 and T4 respectively (Figure 1), heparinized syringes were used to collect 3 mL blood samples for blood gas analysis, via the catheters at the distal extremities of the arteriotomies (superficial femoral artery); and venous blood was drawn by direct puncture of the femoral vein for lactate and CP assays; and, a ruler and a saline column were used to determine blood pressure at distal (superficial femoral) and proximal (common femoral) arterial extremities.

Throughout the study, 5,000 UI of heparin were administrated every 3 hours.

After all procedures had been completed, animals in group 2 were euthanized as directed by Federal Veterinary Medical Council resolution 1000/2012 (Conselho Federal de Medicina Veterinária).

### Statistical analysis

The results were subjected to analysis of normality of distribution using the Anderson Darling test. The chi-square test with Yates’ correction was used to compare qualitative variables, and Student’s *t* test was used for quantitative variables, with statistical significance set at p < 0.05.

## RESULTS

The variables measured in limbs arterialized after a mean ischemia time of 27 minutes and 30 seconds were compared with variables measured in the ischemic limbs and control limbs.

### Arterial flow

Doppler examination of arterial flow in the arterialized ischemic limbs at T0 showed absence of flow in one limb, pulsating venous pattern in three, and pulsating arterial pattern in one; at T1 four limbs with blood flow had pulsating venous flow patterns; at T3, pulsating arterial flow was observed in one and pulsating venous flow in three; at T4, three limbs exhibited pulsating venous flow and two limbs had no blood flow. All ischemic limbs exhibited absence of flow throughout the study.

For the purposes of analysis, only data from arterialized ischemic limbs that had flow detected by Doppler US were considered: four animals up to T3 and three animals at T4.

### Temperature

Although there was no environmental control, the mean variations in temperature, at the extremities in the study group, exhibited similar curves, with means significantly lower to those measured for the control group over time (Table 1). When compared against each other, the mean temperatures for the arterialized ischemic limbs exhibited a non-significant (p= 0.55) difference; temperatures were 2.25 °C higher than the ischemic limbs at T4 (Table 2).

**Table 1:** Comparison of temperatures in arterialized limbs and limbs in ischemia in relation to the control group, at four measurement times.

**Temperature (measurement times)**	**Arterialization**	**Control**	**p**	**Ischemia**	**Control**	**p**
	**Mean (standard deviation)**	**Mean (standard deviation)**		**Mean (standard deviation)**	**Mean (standard deviation)**	
T0	29.81 (3.77)	35.65 (0.55)	0.01	30.37 (3.97)	35.65 (0.55)	0.02
T1	26.34 (4.46)	35.65 (0.55)	0.002	26.32 (3.45)	35.65 (0.55)	0.0005
T3	24.77 (2.62)	35.65 (0.55)	0.0001	24.70 (2.35)	35.65 (0.55)	0.0001
T4	26.74 (5.67)	35.65 (0.55)	0.010	24.49 (2.20)	35.65 (0.55)	0.0001

Student’s *t* test.

**Table 2:** Comparison of temperatures in arterialized ischemic limbs and limbs in ischemia at different measurement times.

**Temperature (measurement times)**	**Arterialization**	**Ischemia**	**p**
	**Mean (standard deviation)**	**Mean (standard deviation)**	
T0	29.81 (3.77)	30.37 (3.97)	0.84
T1	26.34 (4.46)	26.32 (3.45)	0.99
T3	24.77 (2.62)	24.70 (2.35)	0.97
T4	26.74 (5.67)	24.49 (2.20)	0.55

Student’s *t* test.

### Blood gas analysis

The pH of arterialized ischemic limbs and ischemic limbs began at slightly higher levels than the pH of the control group and followed similar curves, with a non-significant fall at T2, a little more accentuated than at T4 (Tables 3, 4, and 5). When compared against each other, they did not exhibit significance differences (Tables 6, 7, and 8).

**Table 3:** Comparison of biochemical variables and arterial blood pressure levels in arterialized limbs and limbs in ischemia in relation to the control group at measurement time T0.

**Variables**	**Arterialization**	**Control**	**p**	**Ischemia**	**Control**	**p**
	**Mean (standard deviation)**	**Mean (standard deviation)**		**Mean (standard deviation)**	**Mean (standard deviation)**	
pH	7.32 (0.11)	7.30 (0.15)	0.83	7.31 (0.12)	7.30 (0.15)	0.92
BE	2.68 (1.02)	2.38 (2.47)	0.83	2.18 (1.38)	2.38 (2.47)	0.89
HCO_3_^-^	29.45 (1.77)	30.62 (4.17)	0.62	29.15 (2.29)	30.62 (4.17)	0.55
pO_2_	166.33 (61.91)	298.10 (128.32)	0.10	158.93 (58.34)	298.10 (128.32)	0.09
pCO_2_	60.75 (16.19)	68.82 (34.49)	0.68	60.60 (17.63)	68.82 (34.49)	0.68
Lactate	18.55 (2.99)	17.14 (6.96)	0.72	21.20 (10.28)	17.14 (6.96)	0.50
CP	1,647.50 (590.89)	940.00 (364.18)	0.06	1,612.50 (563.68)	940.00 (364.18)	0.07
Arterial blood pressure	55.75 (15.76)	35.86 (22.86)	0.18	47.50 (22.49)	35.86 (22.86)	0.47

**Table 4:** Comparison of biochemical variables and arterial blood pressure levels in arterialized limbs and limbs in ischemia in relation to the control group at measurement time T2.

**Variables**	**Arterialization**	**Control**	**p**	**Ischemia**	**Control**	**p**
	**Mean (standard deviation)**	**Mean (standard deviation)**		**Mean (standard deviation)**	**Mean (standard deviation)**	
pH	7.26 (0.08)	7.30 (0.15)	0.65	7.25 (0.06)	7.30 (0.15)	0.55
BE	1.13 (1.97)	2.38 (2.47)	0.44	0.68 (1.41)	2.38 (2.47)	0.26
HCO_3_^-^	29.30 (1.47)	30.62 (4.17)	0.57	29.08 (1.28)	30.62 (4.17)	0.50
pO_2_	143.65 (89.98)	298.10 (128.32)	0.08	167.53 (143.26)	298.10 (128.32)	0.19
pCO_2_	57.80 (23.85)	68.82 (34.49)	0.61	68.40 (11.02)	68.82 (34.49)	0.98
Lactate	40.30 (19.38)	17.14 (6.96)	0.04	36.10 (21.17)	17.14 (6.96)	0.10
CP	1,747.00 (556.34)	940.00 (364.18)	0.03	1,704.25 (554.79)	940.00 (364.18)	0.04
Arterial blood pressure	42.63 (19.61)	35.86 (22.86)	0.65	37.75 (15.76)	35.86 (22.86)	0.89

Student’s *t* test.

**Table 5:** Comparison of biochemical variables and arterial blood pressure levels in arterialized limbs and limbs in ischemia in relation to the control group at measurement time T4.

**Variables**	**Arterialization**	**Control**	**p**	**Ischemia**	**Control**	**p**
	**Mean (standard deviation)**	**Mean (standard deviation)**		**Mean (standard deviation)**	**Mean (standard deviation)**	
pH	7.21 (0.12)	7.30 (0.15)	0.41	7.20 (0.09)	7.30 (0.15)	0.34
BE	-0.50 (3.58)	2.38 (2.47)	0.41	1.07 (2.72)	2.38 (2.47)	0.11
HCO_3_^-^	29.07 (1.61)	30.62 (4.17)	0.57	27.87 (2.48)	30.62 (4.17)	0.35
pO_2_	95.83 (13.82)	298.10 (128.32)	0.04	94.67 (3.89)	298.10 (128.32)	0.04
pCO_2_	76.23 (21.75)	68.82 (34.49)	0.75	71.87 (15.09)	68.82 (34.49)	0.89
Lactate	61.93 (19.70)	17.14 (6.96)	0.003	55.33 (19.15)	17.14 (6.96)	0.006
CP	2,020.67 (621.81)	940.00 (364.18)	0.02	1,879.33 (425.96)	940.00 (364.18)	0.02
Arterial blood pressure	33.17 (19.20)	35.86 (22.86)	0.87	36.67 (13.58)	35.86 (22.86)	0.96

Student’s *t* test.

**Table 6:** Comparison of biochemical variables and arterial blood pressure levels in arterialized limbs and limbs in ischemia at measurement time T0.

**Variables**	**Arterialization**	**Ischemia**	**p**
	**Mean (standard deviation)**	**Mean (standard deviation)**	
pH	7.32 (0.11)	7.31 (0.12)	0.91
BE	2.68 (1.02)	2.18 (1.38)	0.58
HCO_3_^-^	29.45 (1.77)	29.15 (2.29)	0.84
pO_2_	166.33 (61.91)	158.93 (58.34)	0.87
pCO_2_	60.75 (16.19)	60.60 (17.63)	0.99
Lactate	18.55 (2.99)	21.20 (10.28)	0.64
CP	1,647.50 (590.89)	1,612.50 (563.68)	0.93
Arterial blood pressure	55.75 (15.76)	47.50 (22.49)	0.57

Student’s *t* test.

**Table 7:** Comparison of biochemical variables and arterial blood pressure levels in arterialized limbs and limbs in ischemia at measurement time T2.

**Variables**	**Arterialization**	**Ischemia**	**p**
	**Mean (standard deviation)**	**Mean (standard deviation)**	
pH	7.26 (0.08)	7.25 (0.06)	0.85
BE	1.13 (1.97)	0.68 (1.41)	0.72
HCO_3_^-^	29.30 (1.47)	29.08 (1.28)	0.83
pO_2_	143.65 (89.98)	167.53 (143.26)	0.79
pCO_2_	57.80 (23.85)	68.40 (11.02)	0.45
Lactate	40.30 (19.38)	36.10 (21.17)	0.78
CP	1,747.00 (556.34)	1,704.25 (554.79)	0.92
Arterial blood pressure	42.63 (19.61)	37.75 (15.76)	0.71

Student’s *t* test.

**Table 8:** Comparison of biochemical variables and arterial blood pressure levels in arterialized limbs and limbs in ischemia at measurement time T4.

**Variables**	**Arterialization**	**Ischemia**	**p**
	**Mean (standard deviation)**	**Mean (standard deviation)**	
pH	7.21 (0.12)	7.20 (0.09)	0.91
BE	0.50 (3.58)	1.07 (2.72)	0.58
HCO_3_^-^	29.07 (1.61)	27.87 (2.48)	0.52
pO_2_	95.83 (13.82)	94.67 (3.89)	0.89
pCO_2_	76.23 (21.75)	71.87 (15.09)	0.79
Lactate	61.93 (19.70)	55.33 (19.15)	0.70
CP	2,020.67 (621.81)	1,879.33 (425.96)	0.76
Arterial blood pressure	33.17 (19.20)	36.67 (13.58)	0.81

Student’s *t* test.

### Base excess

Both arterialized ischemic limbs and limbs maintained in ischemia exhibited a progressive fall in base excess (BE) in relation to the control group, which was more accentuated in limbs maintained in ischemia (Tables 3, 4, and 5). When arterialized limbs were compared with those maintained in ischemia, there was a non-significant (p=0.58) difference of 1.57 at T4 (Tables 6, 7, and 8).

### Bicarbonate concentration

Both arterialized ischemic limbs and limbs maintained in ischemia exhibited a progressive fall in the concentration of bicarbonate (HCO_3_^-^), which was more accentuated in the ischemic limbs, especially at T4 (Tables 3, 4, and 5), but without significance either in relation to the control group or to the arterialized limbs (Tables 6, 7, and 8).

### Partial oxygen pressure

Partial oxygen pressures (pO_2_) in the arterialized ischemic limbs and the ischemic limbs started from lower levels than the control limbs and followed similar curves, with significant drops at T4 (Tables 3, 4, and 5) Comparisons between them did not reveal significance (Tables 6, 7, and 8).

### Partial carbon dioxide pressure

Partial carbon dioxide pressure (pCO_2_) values were similar and lower than in the control group at T0, followed by a progressive increase in mean pCO_2_ in limbs maintained in ischemia and a mild fall in mean pCO_2_ in the arterialized ischemic limbs at T2. The means for both groups exhibited increases at T4, although the increase was greater in the ischemia with arterialization group (Tables 3, 4, and 5). Comparisons between means were not significant (Tables 6, 7, and 8).

### Lactate

Mean lactate values started from a baseline level similar to that in the control group and exhibited similar curves, with a progressive increase at T2, which was significant for arterialized ischemic limbs, and a more accentuated increase at T4 that was significant both for arterialization and ischemia (Tables 3, 4, and 5). Comparisons between means were not significant (Tables 6, 7, and 8).

### Creatine phosphokinase

Mean CP values started from higher baseline levels than the control group, exhibiting significant increases both in arterialized ischemic limbs and in those in ischemia at T2 and T4 (Tables 3, 4, and 5). However, means did not exhibit significant differences (Tables 6, 7, and 8).

### Arterial blood pressure

The ratios of distal arterial pressures to proximal arterial pressures were used for the purposes of calculations. In arterialized ischemic limbs, the mean of this ratio started from a higher baseline than in the control group and than in limbs in ischemia. The means in both intervention groups exhibited a drop at T2, although the ratio for arterialized ischemic limbs remained higher. At T4, the means approached those in the control group (Tables 3, 4, and 5) and were not significantly different from each other (Tables 6, 7, and 8).

The curves for absolute distal pressures exhibited similar patterns to the ratios, also without significance.

## DISCUSSION

There are no experimental studies of ischemia and reperfusion (I/R) by arterialization of veins that have tested the variables of interest in animals. Our model induced acute ischemia, which is different from the chronic form, in which a period of ischemia without necrosis can lead to production of stimuli for arteriogenesis.

Although both procedures were conducted on the same animal, one on each hind limb, the variables studied were determined separately. Use of the contralateral limb is common in studies of unilateral ischemia,^21^^,^ 22 because it means that the measurements can be taken over the same substrate, although it is not possible to entirely rule out interference from one limb with the other. The experimental model used in this study reproduces the conditions of ischemia and reperfusion by venous arterialization, with retrograde transfer of the higher pressure arterialized venous flow (proximal arteries) to the lower pressure arterial bed (distal arteries).^13^

When Doppler US did not detect flow at the extremity, the data collected from the arterialized ischemic limb were disregarded.

Doppler examination showed pulsating arterial and pulsating venous flow patterns, as are seen in arteriovenous fistulas.^23^

Sasajima et al. studied deep venous arterialization in mice, showing that rupture of the valves at the level of the femoral vein was accompanied by an increase in skin temperature in the region of the hip and thigh and at the knee joint. They also showed that hyperthermia of the distal extremity only occurred when the valves in the popliteal vein were ruptured.^10^

The increased mean temperature of arterialized ischemic limbs, observed from T3 onwards, suggests that the system is patent and confirms the utility of valvulotomy of the arterialized vein.

Tissue edema occurs during reperfusion, with potential exacerbation of tissue damage and the systemic response. In the context of I/R, local and systemic changes involve many different systems: endothelial, circulatory, metabolic, acid-base, etc.^20^

Szokoly et al. demonstrated that in rats subjected to I/R in the hind limbs there was a continuous and significant drop in venous pH compared to baseline in the first hour. This was accompanied by changes in pCO_2_ and pO_2_, which exhibited moderate signs of respiratory compensation.^20^

Mondek et al. reported a pilot study in which the maximum level of acidosis occurred 2 hours after the start of reperfusion to a limb that had been ischemic due to vascular clamping. The blood sample was drawn from the ipsilateral femoral vein.^24^

Findings from this study showed metabolic acidosis, with a drop in the BE value and little change to HCO_3_^-^ The fall in BE suggests buffering, which allows a certain stability in pH levels. The variations in pCO_2_ and pO_2_ were nonspecific, with a significant drop in pO_2_ in both limbs, combined with an increase in pCO_2_ at the end of the experiment. These findings are compatible with other studies of I/R.^20^^,^ 25

Szokoly et al. and Mondek et al. conducted studies in which pH was measured in the collector vein of the limb, because reperfusion was via arterial pathways.^20^^,^ 24 In our study, we conducted blood gas analysis of samples from the distal extremity of the femoral artery, since reperfusion was via venous vessels, and our intention was to evaluate retrograde flow.

During surgery for abdominal aortic aneurysms that involved I/R of extremities, Sako et al. observed transitory increases in lactate and a reduction in pH in iliac veins after reperfusion.^26^

Theodoraki et al. studied the transhepatic lactate gradient during I/R in hepatectomies and observed increased hepatic lactate production 50 minutes after reperfusion. They also demonstrated a positive correlation between intraoperative systemic lactate levels and the transhepatic lactate gradient, suggesting that hepatic reperfusion made a significant contribution to the systemic hyperlactatemic state.^27^

The mean lactate values observed in the intervention groups in this study started from a similar baseline level to the control group and exhibited similar curves, with a progressive and significant increase that was more accentuated in arterialized ischemic limbs, in which reperfusion was retrograde (flow present on Doppler US).

Woodruff et al. conducted a study to evaluate the capacity of a drug to counter injury provoked by ischemia and subsequent reperfusion (I/R), demonstrating increases in CP in the group subjected to I/R, which were not observed in a group that was only subjected to ischemia.^28^ This may suggest that the pathogenic basis of the increase in the marker of muscle injury could be reperfusion after ischemia.

In this study, CP values exhibited similar behavior to lactate levels, showing significant increases at T2 and even more accentuated increases at T4, especially in the arterialized ischemic limbs.

Szokoly et al. studied I/R in the hind legs of mice, observing a fall in mean arterial blood pressure of around 20% after reperfusion. Possible compensatory events parallel to the procedure or even vasodilation due to reperfusion of the limb could explain the drop in pressure.^20^

The mean ratio of arterial pressures in arterialized ischemic limbs started from a higher level than the control group and than the limbs in ischemia. Means in both intervention groups exhibited a reduction at T2, although the arterialized ischemic limbs maintained a higher level. At T4, means approached the means for the control group. The curve for the fall in distal arterial blood pressure was similar to the curves for the ratio between distal and proximal arterial blood pressures, which is not suggestive of interference in the values from proximal arterial blood pressure.

## CONCLUSIONS

The findings of this study are compatible with metabolic acidosis, with significant increases in CP and lactate, suggesting cellular damage in both limbs and signs of retrograde reperfusion due to maintenance of flow, as seen on Doppler US, in the arterialized ischemic limbs.
